# ﻿*Rungiafangdingiana* (Acanthaceae), a new species from Guangxi, China

**DOI:** 10.3897/phytokeys.202.86653

**Published:** 2022-07-28

**Authors:** Zhe-Li Lin, Yun-Hong Tan, Yun-Feng Huang, Yun-Fei Deng

**Affiliations:** 1 Key Laboratory of Plant Resources Conservation & Sustainable Utilization, South China Botanical Garden, Chinese Academy of Sciences, Guangzhou, 510650, China South China Botanical Garden, Chinese Academy of Sciences Guangzhou China; 2 Center for Integrative Conservation, Xishuangbanna Tropical Botanical Garden, Chinese Academy of Sciences, Menglun, Mengla, 666303, Yunnan, China Tropical Botanical Garden, Chinese Academy of Sciences Menglun China; 3 Southeast Asia Biodiversity Research Institute, Chinese Academy of Sciences, Yezin, Nay Pyi Taw 05282, Myanmar Research Institute, Chinese Academy of Sciences Nay Pyi Taw Myanmar; 4 Guangxi Institute of Traditional Medical and Pharmaceutical Sciences, No. 20-1, Dongge Lu, Qingxiu Qu, Nanning, 530022, Guangxi, China Guangxi Institute of Traditional Medical and Pharmaceutical Sciences Nanning China; 5 Center of Conservation Biology, Core Botanical Gardens, Chinese Academy of Sciences, Guangzhou, 510650, China Core Botanical Gardens, Chinese Academy of Sciences Guangzhou China

**Keywords:** limestone, SEM, taxonomy, tricolporate pollen

## Abstract

*Rungiafangdingiana*, a new species of Acanthaceae from Guangxi, China is described and illustrated. This new species belongs to Rungiasect.Rungia, and resembles *R.sinothailandica* and *R.burmanica* in the erect perennial herbaceous habit, elliptic leaves and inflorescence form, but differs mainly by the indumentum and the morphology of the bracts and corolla. The pollen and seed micromorphology of this new species are studied, with photographs and a line drawing provided.

## ﻿Introduction

*Rungia* Nees is a genus of Acanthaceae, comprising about 50 species and distributed through tropical and subtropical regions of the Old World (Mabberley 2017). It is closely related to *Justicia* L., but differs from the latter mainly by the rising placenta in ripe fruit (Hansen 1989; Hu 2002; Hu et al. 2011; Kiel et al. 2017; Deng 2020; Deng and Gao 2020). Some *Rungia* species were transferred to *Justicia* in regional Floras (Darbyshire et al. 2010; Wood 2014; Vollesen 2015), but the recent systematic studies (Kiel et al. 2017; Manzitto-Tripp et al. 2021) do not agree with this treatment based on molecular and some morphological evidence. In this work, we treat *Rungia* as a separate genus; however, the relationship between *Rungia* and its related genera is awaiting further study.

Sixteen species of *Rungia* were recognized in the “Flora of China” (Hu et al. 2011). Amongst these, *R.monetaria* (Benoist) B. Hansen was misidentified (the collection is actually *R.flaviflora*) and should be excluded from the list of species found in China, *R.axilliflora* and *R.densiflora* were treated as synonyms of *R.stolonifera* (Lin et al. 2020), Whereas *R.burmanica* (C. B. Clarke) B. Hansen is newly recorded in China (Lin and Deng 2017a, 2018), and recently two new species, *Rungiasinothailandica* Z. L. Lin & Y. F. Deng and *R.flaviflora* Z. L. Lin & Y. F. Deng, were described (Lin and Deng 2017b, 2018). Consequently, sixteen species of *Rungia* are still recognized in China at present.

During examination of the specimens in Herbaria (HITBC, PE), a distinctive specimen (*H. Wang 6616*) collected from Napo County, Guangxi in 2002, attracted our attention because of its secund spike, which is the typical character of the genus *Rungia*, but it lacked flower and fruit. Recently, it was collected again in the field complete with flowers and fruits from Napo in 2015 (*Y. H. Tan et al. 4366*) and 2019 (*Y. F. Deng et al. 29030*). After careful study of the specimens and living plants, we confirmed that it represents a new species of *Rungia* and is described below.

## ﻿Materials and methods

The morphological description of the new species was based on both fresh and dried materials. The voucher specimens (*Y. H. Tan et al. 4366*, *Y. F. Deng et al. 29030* and *H. Wang 6616*) are deposited in the Herbaria of South China Botanical Garden, Chinese Academy of Sciences (**IBSC**), Xishuangbanna Tropical Botanical Garden, Chinese Academy of Sciences (**HITBC**) and Institute of Botany, Chinese Academy of Sciences (**PE**).

The pollen and seeds were washed in 70% alcohol and then gilded using the auto-fine sputter coater (JEOL JFC-1600 Auto Fine Coater, Japan). The micrographs were taken using the scanning electron microscope (JEOL Model JSM-6360 LV, Japan). The polar (P) axis and equatorial (E) diameter of 20 pollen grains were measured and the average, maximum, minimum and ratio (P/E) values were recorded to represent the range of variation. The pollen terminology follows Daniel (1998) and Scotland and Vollesen (2000). The seed terminology follows Graham (1988).

## ﻿Taxonomic treatment

### ﻿ Rungia fangdingiana

Taxon classificationPlantaeLamialesAcanthaceae

Z. L. Lin, Y. F. Deng & Y. H. Tan sp. nov.

2F08DBC6-78AD-58A6-AEA5-81AA0201EF44

urn:lsid:ipni.org:names:77302500-1

Figs 1 , 2 , 3

#### Type.

China. Guangxi Province: Napo County, Baisheng Xiang, Nongmiao Cun, 965 m elev., 24 July 2015, *Y. H. Tan et al. 4366* (holotype: IBSC!; isotypes: IBSC!, HITBC!).

#### Diagnosis.

Similar to *Rungiasinothailandica* and *R.burmanica*, but is readily distinguishable by the flat, entire bract margin (vs. crispate, tawny membranous bract margin in *R.sinothailandica*, and crenulate bract margin in *R.burmanica*), glabrous calyx and capsule (vs. puberulous calyx and puberulent capsule in *R.sinothailandica* and *R.burmanica*), pale yellow corolla with red stripes, upper lip unlobed (vs. white corolla with red stripes, upper lip 2-lobed in *R.sinothailandica*, and white corolla with purple dotted stripes, upper lip unlobed in *R.burmanica*). A detailed comparison of the three species is given in Table 1.

**Table 1.** Morphological comparison of *Rungiafangdingiana*, *R.sinothailandica* and *R.burmanica*.

Characters	*R.fangdingiana*	*R.sinothailandica*	*R.burmanica*
**Stem**	glabrous	bifariously pubescent	glabrous
**Leaves**	glabrous	pubescent	glabrous
**Sterile bract**	without membranous margin, margin entire	membranous margin crispate, hyaline with slightly tawny colour, 1.5 mm wide	without membranous margin, margin crenulate towards the apex
**Fertile bract**	rhombic to elliptic, membranous margin flat and hyaline, 0–0.5 mm wide, margin entire	obovate to elliptic, membranous margin crispate, hyaline at base and tawny at apex, 2 mm wide	obovate, without membranous margin, margin crenulate towards the apex
**Calyx**	lobes linear, glabrous	lobes linear, puberulous	lobes linear, puberulous
**Corolla**	pale yellow with red stripes, upper lip unlobed	white with red stripes, upper lip 2-lobed	white with purple dotted stripes, upper lip unlobed
**Capsule**	glabrous	puberulent	puberulent

**Figure 1.**
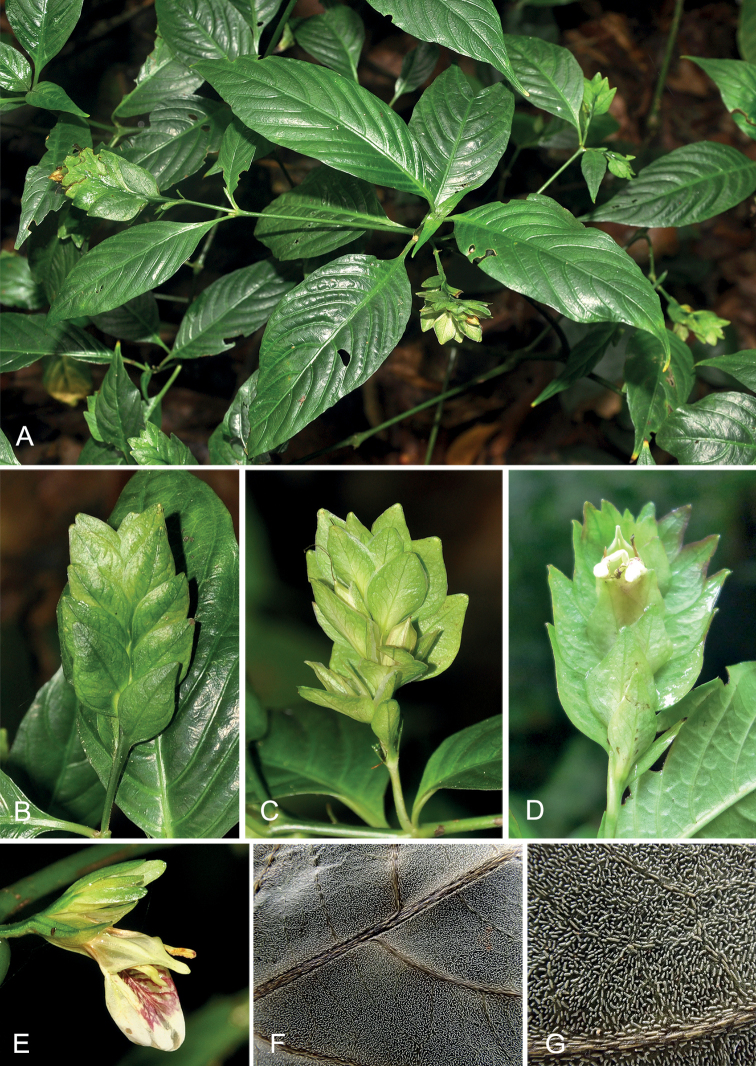
Photographs of *Rungiafangdingiana* sp. nov. **A** habit **B** spike (abaxial view showing the sterile bracts) **C** spike (adaxial view showing the fertile bracts and fruits) **D** spike (adaxial view showing the fertile bracts and corolla) **E** corolla **F, G** adaxial view of leaf blade (showing the linear cystoliths) **A–E** by Yun-Hong Tan, **F**, **G** by Zhe-Li Lin.

#### Description.

Perennial erect herb, about 1 m tall. Stem terete, glabrous. Leaves usually anisophyllous; petiole 1–2 cm long, glabrous; blade elliptic, 5–15 × 2–5 cm, base cuneate, margin entire, apex attenuate or acute, both surfaces glabrous, but densely covered with linear cystoliths, secondary veins 5–10 on each side of mid-vein. Inflorescence of terminal and axillary spikes, 3–7 cm long; peduncle 1–2 cm long, glabrous. Bracts 4-ranked, only two ranks fertile; sterile bracts ovate, oblique at base, 1.4–1.6 × 0.9–1.1 cm, green, glabrous, both surfaces covered with linear cystoliths, lacking hyaline margins; fertile bracts rhombic to elliptic, symmetrical, 1.3–1.5 × 0.9–1.1 cm, green, glabrous, both surfaces covered with linear cystoliths, membranous hyaline margin flat, 0–0.5 mm wide. Bracteoles elliptic, 1.0–1.1 × 0.3–0.4 cm, green, glabrous, abaxial surface covered with linear cystoliths, membranous hyaline margin flat, sometimes slightly puberulent at margin, ca. 1 mm wide. Calyx 5-lobed almost to the base, lobes linear, equal, 8–8.5 × 0.9–1.1 mm, glabrous. Corolla bilabiate, glabrous, ca. 1.5 cm long, pale yellow with red stripes on lower lip and throat; tube cylindrical at base and enlarged abruptly in throat, ca. 1 cm long; upper lip unlobed, attenuate at apex; lower lip 3-lobed, lobes rounded at apex. Stamens 2, 4.5–5 mm long, inserted at base of corolla throat, base adnate with corolla tube; filaments glabrous; anthers bithecous, superposed, ca. 3 mm long, thecae ovoid, glabrous, lacking a conspicuous basal spur. Pollen prolate, 35.99 (33.2–38.5) × 18.94 (15.7–21.6) μm, with P/E = 1.90, tricolporate, with one row of insulae on each side of aperture, exine ornamentation reticulate. Ovary glabrous, ca. 1.5 mm long; style slightly puberulent at base and middle part, ca. 1 cm long; stigma minutely 2-lobed. Capsule clavate, stipitate, glabrous, 1.1–1.2 × 0.3–0.35 cm. Seeds suborbicular to elliptic, compressed, dark brown, 2.5–3.5 × 2–2.5 mm, surface covered with brain-like verrucae.

**Figure 2.**
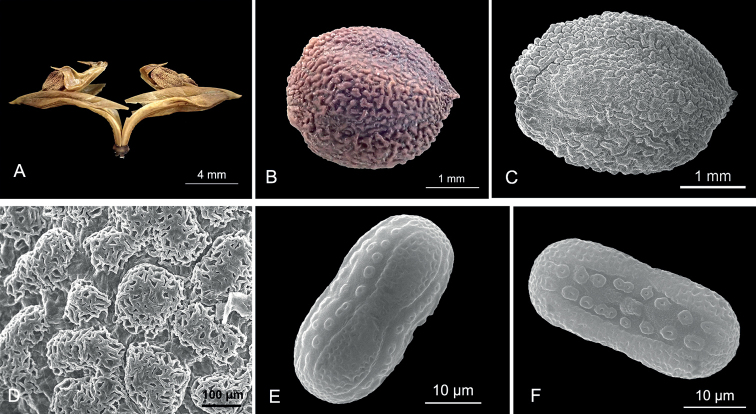
Fruit, seed and pollen morphology of *Rungiafangdingiana* sp. nov. **A** dehiscent capsule (showing the rising placenta and seeds) **B** seed **C** seed testa (SEM) **D** detail of the verrucae of seed testa (SEM) **E** interapertural view of pollen grain **F** apertural view of pollen grain. Photos by Zhe-Li Lin.

#### Phenology.

It was observed with flower and fruit at the same time from October to December and June to July.

#### Etymology.

This new species is named in honor of Mr. Fang Ding (1924–2017) for his contributions to studies of the family Acanthaceae from Guangxi, China. He was the co-author of the account of family Acanthaceae for “Flora of Guangxi” and has published 37 new taxa of Acanthaceae from Guangxi (Fang and Deng 2017).

#### Vernacular name.

方鼎孩儿草 (Chinese pinyin: fāng dǐng hái ér cǎo).

#### Distribution and habitat.

This species is currently known from three localities in Napo County of Guangxi Province. It grows close to stream sides in evergreen forest on limestone at elevations of 600 to 1200 m.

#### Conservation status.

During the field investigations, only three scattered populations of *Rungiafangdingiana* were discovered, which all grow in forest on limestone in Napo county. Because of its narrow distribution (extent of occurrence < 20,000 km^2^), limited locations (< 10), with an estimated population size of < 1000 mature individuals, and there is continuing decline estimated in quality of habitat and number of locations due to human activities as the localities are not protected, it should be assessed as Vulnerable (VU) (B1ab(iii)(iv)) according to the IUCN Red List Categories and Criteria (IUCN 2012, 2022). However, the area around the localities of this new species is poorly investigated, and further rigorous investigations are necessary to confirm this assessment.

**Figure 3.**
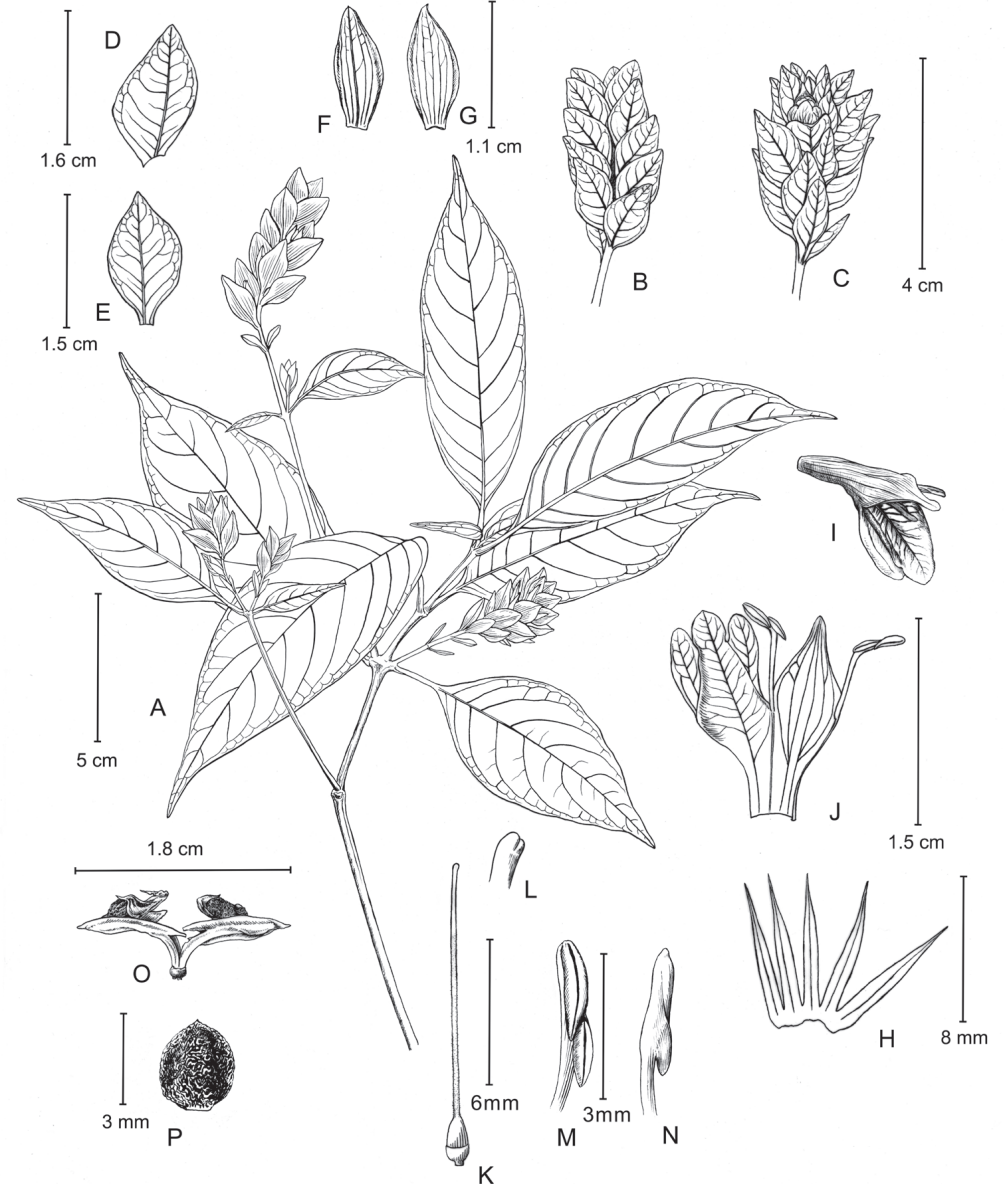
Line drawings of *Rungiafangdingiana* sp. nov. **A** flowering branch **B** spike (abaxial view showing the sterile bracts) **C** spike (adaxial view showing the fertile bracts and corolla) **D** sterile bract **E** fertile bract **F** bracteole (abaxial view) **G** bracteole (adaxial view) **H** calyx **I** corolla **J** opened corolla **K** pistil **L** stigma **M** stamen (adaxial view) **N** stamen (abaxial view) **O** dehiscent capsule (showing the rising placenta and seeds) **P** seed. Drawn by Yun-Xiao Liu.

#### Additional specimens examined

**(*paratypes*)**: China. Guangxi, Napo County: Nonghe Cun, on the way from Nonghe Cun to Tongziwan Power Station, on the rocks near stream-sides, 674 m elev., 11 December 2019, *Y. F. Deng et al. 29030* (IBSC!); Napo County, in limestone forest, 1200 m elev., 24 October 2002, *H. Wang 6616* (HITBC!, PE!).

## ﻿Discussion

*Rungiafangdingiana* fits well with the circumscription of Rungiasect.Rungia according to the infrageneric classification by Gao and Deng (2007), as its flowers are arranged in a secund spike with heteromorphic bracts in two ranks of fertile bracts and two ranks of sterile bracts. Among the species in Rungiasect.Rungia, this new species is morphologically most similar to *Rungiasinothailandica* and *R.burmanica*, a key to these three similar species are provided as below.

### ﻿Key to *Rungiafangdingiana* and morphologically similar species in China

**Table:** 

1	Bracts with a crispate, tawny margin, upper lip of corolla 2-lobed	***R.sinothailandica***
–	Bracts lacking a crispate, tawny margin, upper lip of corolla unlobed	**2**
2	Calyx and capsule glabrous; corolla pale yellow with red stripes	***R.fangdingiana***
–	Calyx and capsule puberulous; corolla white with purple dots	***R.burmanica***

Previously, the pollen morphology of ten species were reported in *Rungia* (Raj 1961, 1965; Scotland and Vollesen 2000; Rueangsawang et al. 2013; Lin et al. 2016; Lin and Deng 2017b, 2018; Kiel et al. 2017), amongst which nine species have dicolporate pollen grains and one species (*Rungiaflaviflora*) has tricolporate pollen grains. In this study, *R.fangdingiana* is the second species found to have tricolporate pollen grains in *Rungia*.

The seed micromorphology of the four species in *Rungia* has been reported previously (Rueangsawang et al. 2012; Lin et al. 2016; Kiel et al. 2017; Lin and Deng 2017b), with two species having volcano-like verrucae on the seed testa, one species having brain-like verrucae, and *R.repens* was not observed under SEM (Kiel et al. 2017) so the detail of verrucae is not clear. The seed testa of *R.fangdingiana* has brain-like verrucae.

The micromorphology of pollen and seed is important in taxonomy in the family Acanthaceae (Radlkofer 1883; Lindau 1895; Raj 1961; Graham 1988; Daniel 1998; Scotland and Vollesen 2000; Cui and Hu 2005; Hu et al. 2005a, 2005b; Rueangsawang et al. 2012, 2013; Kiel et al. 2017; Manzitto-Tripp et al. 2021). However, due to the limited number of species studied in *Rungia*, further studies on pollen and seed morphology are necessary to understand its significance in species delimitation and the systematics in this genus.

## Supplementary Material

XML Treatment for Rungia fangdingiana
